# Characterization of HIV-Specific CD4+ T Cell Responses against Peptides Selected with Broad Population and Pathogen Coverage

**DOI:** 10.1371/journal.pone.0039874

**Published:** 2012-07-05

**Authors:** Marcus Buggert, Melissa M. Norström, Chris Czarnecki, Emmanuel Tupin, Ma Luo, Katarina Gyllensten, Anders Sönnerborg, Claus Lundegaard, Ole Lund, Morten Nielsen, Annika C. Karlsson

**Affiliations:** 1 Division of Clinical Microbiology, Department of Laboratory Medicine, Karolinska Institutet, Stockholm, Sweden; 2 HIV and Human Genetics, National Microbiology Laboratory, Public Health Agency of Canada, Winnipeg, Canada; 3 Department of Virology, Swedish Institute for Infectious Disease Control, Stockholm, Sweden; 4 Department of Medical Microbiology, University of Manitoba, Winnipeg, Canada; 5 Gay Men’s Health Clinic, Stockholm South General Hospital (Södersjukhuset), Stockholm, Sweden; 6 Division of Infectious Diseases, Department of Medicine Huddinge, Karolinska Institutet, Stockholm, Sweden; 7 Center for Biological Sequence Analysis, Department of Systems Biology, Technical University of Denmark, Lyngby, Denmark; University of Cape Town, South Africa

## Abstract

CD4+ T cells orchestrate immunity against viral infections, but their importance in HIV infection remains controversial. Nevertheless, comprehensive studies have associated increase in breadth and functional characteristics of HIV-specific CD4+ T cells with decreased viral load. A major challenge for the identification of HIV-specific CD4+ T cells targeting broadly reactive epitopes in populations with diverse ethnic background stems from the vast genomic variation of HIV and the diversity of the host cellular immune system. Here, we describe a novel epitope selection strategy, *PopCover,* that aims to resolve this challenge, and identify a set of potential HLA class II-restricted HIV epitopes that in concert will provide optimal viral and host coverage. Using this selection strategy, we identified 64 putative epitopes (peptides) located in the Gag, Nef, Env, Pol and Tat protein regions of HIV. In total, 73% of the predicted peptides were found to induce HIV-specific CD4+ T cell responses. The Gag and Nef peptides induced most responses. The vast majority of the peptides (93%) had predicted restriction to the patient’s HLA alleles. Interestingly, the viral load in viremic patients was inversely correlated to the number of targeted Gag peptides. In addition, the predicted Gag peptides were found to induce broader polyfunctional CD4+ T cell responses compared to the commonly used Gag-p55 peptide pool. These results demonstrate the power of the *PopCover* method for the identification of broadly recognized HLA class II-restricted epitopes. All together, selection strategies, such as *PopCover,* might with success be used for the evaluation of antigen-specific CD4+ T cell responses and design of future vaccines.

## Introduction

The cellular immune response represents a crucial component of the battle against viral infections, including that of human immunodeficiency virus type 1 (HIV). CD4+ T cells are critical for maintaining and mobilising the adaptive immune response, essentially providing helper functions to different arms of the response. For instance, CD4+ T cells govern memory B cell generation [1] and CD8+ T cell memory maintenance [2], [3], migration [4] and mobilisation [5] *in vivo*. In addition, the borderline protection provided by the canarypox-gp120 combination in the RV144 vaccine trial [6] called further attention to HIV-specific CD4+ T cell responses as one of few possible correlates of vaccine protection.

CD4+ T cells direct the response against only a few of the many potential epitopes in a process termed immunodominance. Comprehensive analyses have shown that Gag-specific CD4+ T cell responses dominate the total pool of HIV-specific CD4+ T cells [7], [8], and increased frequencies of these cells have been associated with lower viral loads in HIV-infected individuals [8], [9].

Many thousand different HIV strains have been characterized. Likewise, the restricting element of the CD4+ T cell response consists of the highly variable HLA class II molecules with more than 2000 allelic variants known. This very high pathogen and host diversity imposes a major challenge when it comes to characterization of the HIV-specific CD4+ T cell responses and identification of broadly recognized HLA class II-restricted epitopes in populations with diverse ethnic background. To manage these obstacles, several computational methods have been proposed [10]–[13]. However, most of these methods only partially resolve the issues raised and focus either on the pathogen or the HLA diversity. For instance, the Mosaic Vaccine Tool Suite [11] constructs a mosaic protein sequence so that most viral strains are covered ignoring the aspects of the HLA diversity. On the other hand, the Hotspot Hunter method [12] identifies peptides with promiscuous binding to multiple HLA molecules ignoring the HIV genomic diversity.

We have previously identified broadly reactive HIV-specific CD8+ T cell responses against multiple predicted HLA class I restricted peptides in a cohort infected with various HIV subtypes [14], [15]. In the current study, we developed a novel algorithm, *PopCover*, that takes into consideration both host and viral variability to identify HIV-Gag, -Pol, -Env, -Nef and -Tat peptides restricted to multiple HLA-DR and –DQ alleles. Combining the *PopCover* algorithm with state-of-the-art HLA class II binding prediction, we identified a small set of peptides that in concerts provide coverage of both the viral and host genetic variations in HIV infected patients with diverse ethnic background. Next, by analysing the functional properties of the identified peptides, we were able to recapitulate previous comprehensive findings such as a correlation of viral load to the breadth and polyfunctionality of the HIV-peptide-specific CD4+ T cell responses.

## Results

To enable the identification of broadly reactive HLA class II-restricted peptides, and the patterns of immunodominance and functionality, a diverse patient cohort infected with multiple viral subtypes was identified. The study cohort consisted of 38 HIV-infected individuals infected with ten different HIV-1 subtypes (Table 1). In total, 13 subjects were untreated, and 25 subjects were on treatment. One treated patient had low-grade viremia due to poor treatment adherence. High-resolution HLA-typing for the HLA class I and II genes of 37 patients confirmed that the cohort was highly genetically diverse (data not shown).

**Table 1:** Clinical data of study cohort.

ID	Age	Ethnicity	Sex	CD4+count	Nadir CD4+	Viral load	Subtype^1^	Treatment^2^
THS-1	51	Caucasian	Male	477	120	<50	B	3TC, ABC, ATV, RAL
THS-2	48	Caucasian	Male	768	190	<50	B	EFV, FTC, TDF
THS-3	50	Caucasian	Female	790	120	<50	B	3TC, ABC, NVP
THS-4	51	Caucasian	Male	636	300	<50	B	ETR, RAL
THS-5	52	Caucasian	Male	1495	280	<50	CRF01_AE	EFV, FTC, TDF, RAL
THS-6	51	Latin	Female	576	190	<50	A2	EFV, FTC, TDF
THS-7	59	Caucasian	Male	438	354	<50	CRF01_AE	3TC, ABC, ATV, RTV
THS-8	64	Caucasian	Male	602	273	<50	B	3TC, ABC, EFV
THS-9	44	Caucasian	Male	740	204	<50	CRF01_AE	3TC, ABC, ATV
THS-10	46	Caucasian	Male	500	31	<50	B	EFV, FTC, TDF
THS-11	48	Caucasian	Male	853	576	<50	B	EFV, FTC, TDF
THS-12	48	Caucasian	Male	685	216	<50	C	ATV, FTC, RTV, TDF
THS-13	52	Caucasian	Male	1076	444	<50	B	3TC, ABC, ATV
THS-14	34	Caucasian	Male	822	206	<50	B	3TC, ABC, ATV
THS-15	33	African	Female	660	145	227	A1,G	3TC, TDF, AZT
THS-16	65	Caucasian	Male	686	260	<50	B	3TC, ABC, ATV
THS-17	40	Caucasian	Male	625	10	<50	ND^3^	3TC, ABC, LPV, RAL, RTV
THS-18	54	Caucasian	Male	1010	393	<50	B	3TC, ABC, EFV
THS-19	46	Caucasian	Male	646	367	<50	B	ABC, 3TC, RAL
THS-20	47	Caucasian	Male	900	300	<50	B	3TC, ABC, ATV
THS-21	56	Caucasian	Male	446	284	<50	B	EFV, FTC, TDF
THS-22	47	Caucasian	Male	440	390	<50	B	EFV, FTC, TDF
THS-23	40	Caucasian	Female	364	258	<50	CRF01_AE	EFV, FTC, TDF
THS-24	41	Caucasian	Male	724	170	<50	B	FTC, LPV, RTV, TDF
THS-25	38	Caucasian	Male	780	50	<50	B	3TC, ABC, ATV, RTV
THS-26	37	African	Female	670	447	485	CRF21_A2D	Naive^4^
THS-27	44	Caucasian	Female	600	307	60500	CRF02_AG	Naive
THS-28	30	Caucasian	Female	560	418	2640	CRF03_AB	No treatment^5^
THS-29	62	Caucasian	Male	480	480	27500	CRF01_AE	Naive
THS-30	53	Caucasian	Male	310	310	10100	CRF03_AB	Naive
THS-31	57	Caucasian	Male	475	378	8790	B	Naive
THS-32	35	Caucasian	Male	522	389	37600	B	Naive
THS-33	43	Caucasian	Male	481	403	40500	B	Naive
THS-34	22	Caucasian	Male	534	407	7140	B	Naive
THS-35	24	Latin	Male	460	359	128000	G	Naive
THS-36	45	African	Female	480	400	17800	A1	Naive^4^
THS-37	44	Caucasian	Male	593	593	26900	B	Naive
THS-38	30	Caucasian	Male	410	400	45000	B	Naive

1 The HIV subtypes were obtained through sequence analysis of HIV *gag* (p17 and p24).

2 3TC, lamivudine; ABC, abacavir; ATV, atazanavir; EFV, efavirenz; ETR, etravirine; FTC, emtricitabine; NVP, nevirapine; LPV, lopinavir; RAL, raltegravir; RTV, ritonavir; TDF, tenofovir.

3 Not determined.

4 Female who has received AZT during delivery.

5 Received antiretroviral treatment for only five month, more than a year prior to sample collection.

### Prediction of Broadly Reactive HLA Class II-restricted HIV Peptides

**Figure 1:**
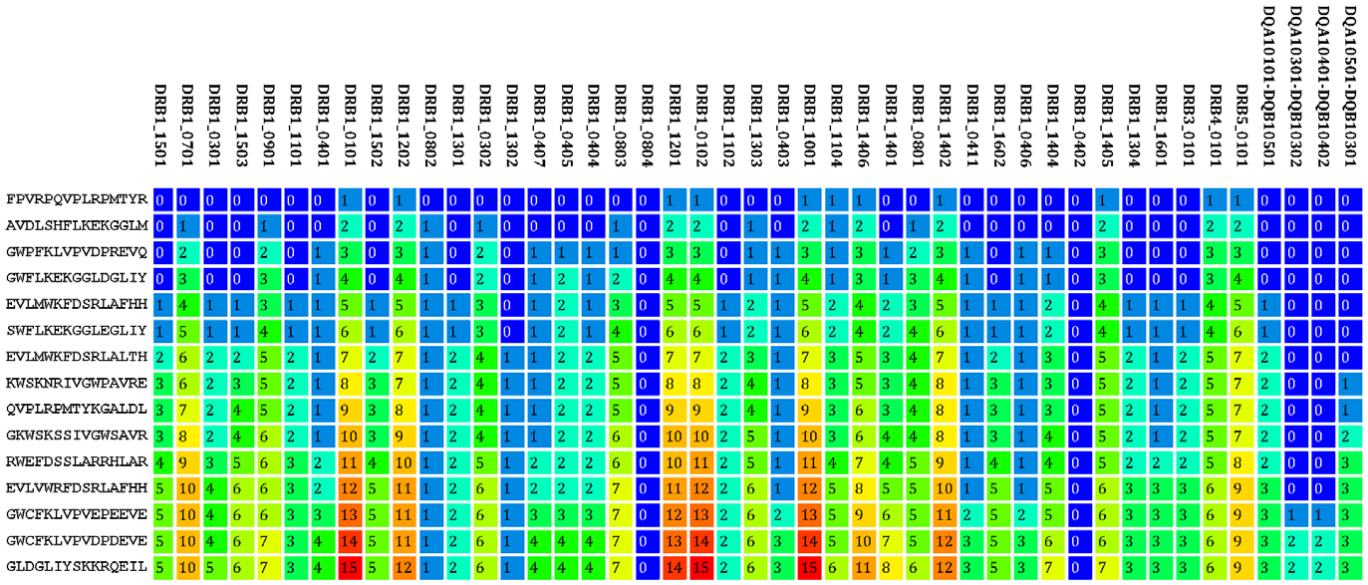
Selection of Nef-specific epitopes using the *PopCover* algorithm. Fifteen Tat 15-mer peptides were selected from the pool of 20,962 predicted HLA class II binders. Each row in the figure represents one peptide, and each column represents an HLA class II molecule. The value in each cell gives the cumulative number of times a given allele was predicted to bind any of the selected peptides. The first selected peptide covered only 11 over the 45 HLA alleles, however, when combined with the other peptides in the pool, the HLA coverage increased to 96% (43 out of 45). When taking the allelic frequency into account, this corresponded to a population-coverage of 99.8%. The coverage of the HIV genomes was similar. The first selected peptide (FPVRPQVPLRPMTYR) is present in 253 of the 396 genomes (64%) included in the analysis. With the other peptides in the pool, this coverage is increased to 99.0%.

A set of full-length HIV proteomes were scanned using *NetMHCII* [16] and *NetMHCIIpan* [17], [18] to identify 15-mer peptides within Gag, Pol, Env, Nef, and Tat that were predicted to bind one or more of 45 prevalent HLA class II alleles. From the pool of more than 225,000 predicted binders, a final set of 64 peptides (15 Gag, 15 Pol, 15 Env, 15 Nef, and 4 Tat) was selected using the *PopCover* algorithm to ensure broad coverage of both the prevalent HLA class II alleles and the major HIV subtypes (see Materials and Methods for details). The power of the *PopCover* selection algorithm can be illustrated from the peptide selection within the Nef protein dataset (Figure 1). This dataset consists of 20,962 unique 15-mer peptides with predicted binding to one or more of the 45 HLA class II alleles. The 15 selected Nef peptides target 43 of the 45 HLA alleles (95%) and 392 of the 396 HIV genomes (99%). Using a selecting strategy in which only the allelic frequency is included in the scoring function (*i.e.,* ignoring *E_ik_* in the scoring function for peptide selection and solely focusing on peptide conservation), these values are reduced to 60% and 92%, respectively. The high coverage of the HLA alleles as a function of peptide selection demonstrates that the *PopCover* selection strategy ensures a broad coverage of the 45 HLA class II alleles. Not one single peptide achieves an HLA coverage beyond 68% (31 alleles) and an HIV genomic coverage beyond 64% (253 strains). Only when taken as a group complementing the individual peptide-characteristics does the peptide-selection reach the high coverage values. The same principle, as described above for Nef, was used for the other HIV protein regions to select high-scoring peptides that generate broadly reactive HIV-specific CD4+ T cell responses. The exact number of peptides included from each HIV region (15 from Gag, Pol, Env, and Nef and 4 from Tat) was chosen to ensure a reasonably homogeneous sampling of the relevant proteomic regions reflecting the protein size and importance for the study.

The overall characteristics of the five peptide-pools in terms of predicted binding properties, HLA restrictions, and the number of targeted HIV strains are given in Table 2. The high genomic diversity of HIV and the variation between the different HIV proteins are quantified in the table. The Env protein, for instance, has the largest number of unique predicted peptide binders. Taking into account the different data set sizes and calculating the number of unique binders per amino acid in each data set, the Env data set has on average 0.346 unique predicted binding peptides per amino acid. The numbers in Table 2 hence confirm that among the five HIV proteins, Env is the most variable followed by Nef, and Pol being the most conserved.

**Table 2:** Characteristics of peptides and peptide-pools for the five HIV protein regions.

Protein	Unique predicted binders	Strains	Protein length	Unique/(length*strain)
**Gag**	31,848	396	498	0.161
**Pol**	42,749	396	1,003	0.108
**Env**	125,926	424	859	0.346
**Nef**	20,962	396	204	0.259
**Tat**	5,608	395	101	0.141

The column “Unique predicted binders” gives the number of unique 15mer peptides in each genomic data set predicted to bind one or more of the 45 HLA class II molecule. Unique/(length*strain) gives the number of unique binders divided with the total number of amino acids in the given genomic data set (protein length * number of strains).

Table 3 demonstrates that all peptide pools, with the exception of the Tat pool, have very similar properties in terms of predicted HLA binding, and the coverage of HLA molecules and viral strains. Due to the relative high sequence conservation of the Pol proteins, the individual Pol peptides targets a higher fraction of the HIV strains than peptides from the other pools. Likewise, as a result of the high sequence variation of the Nef protein data set and the short length of the Nef protein region, the average fraction of HIV strains targeted by the Nef peptide pool is relatively low.

**Table 3:** Characteristics of peptides and peptide-pools for the five HIV protein regions.

		Per pool		Average per peptide		
Protein	Pool size	Fraction of HLAs hit	Fraction of strains hit	1-log50 k	Fraction strains hit	Fraction of HLAs hit
**Gag**	15	0.978	0.997	0.590	0.674	0.578
**Pol**	15	1.000	1.000	0.547	0.891	0.511
**Env**	15	0.978	1.000	0.556	0.531	0.578
**Nef**	15	0.956	0.990	0.545	0.235	0.378
**Tat**	4	0.667	0.775	0.515	0.258	0.356

### Immunodominance of Broadly Reactive HLA Class II-restricted Gag and Nef Peptides

**Figure 2:**
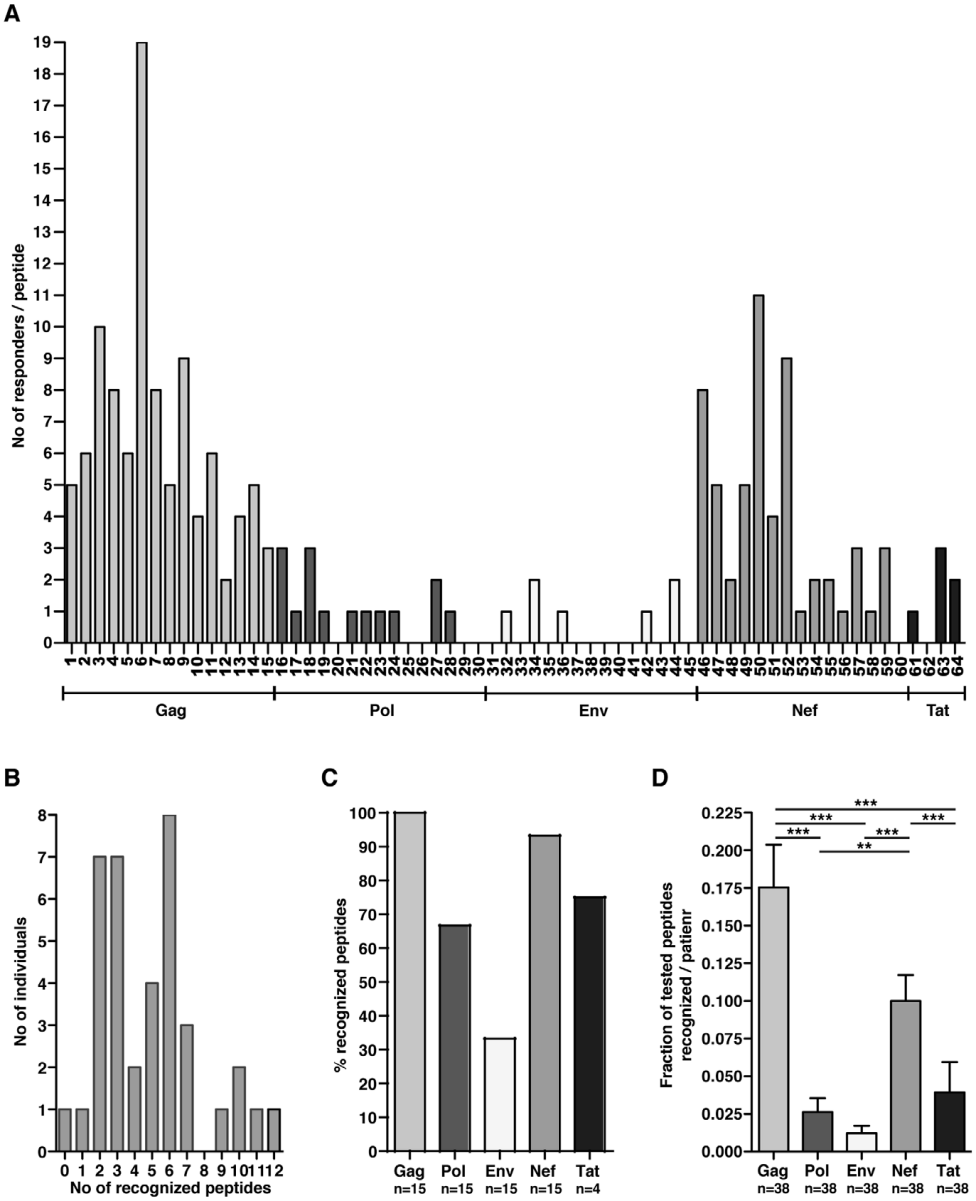
Immunodominance of predicted Gag- and Nef-specific CD4+ T cell responses. (A) Overall immunogenic analysis illustrating the individual peptides that generated a CD4+ T cell response. The vertical axis represents the total number of individuals that recognised each peptide, while the horizontal axis illustrates the individual peptide numbers within each HIV region. (B) Distribution of the number of recognised peptides per individual. (C) Percentage of peptides from different HIV proteins that induced a CD4+ T cell response. (D) Fraction of tested peptides from different HIV protein regions that generated CD4+ T cell responses per individual. The statistical analysis was performed with a one-way ANOVA and a non-parametric Kruskal Wallis test with Dunn’s multiple comparison test to compare all of the pairs of columns; *P<0.05, **P<0.01 and ***P<0.001. The data are derived from 38 independent experiments (mean and SEM).

Peptide-specific T cell responses were first identified by the IFNγ ELISPOT assay using freshly isolated PBMCs. By dividing the peptides into different pools (Table S1), spot forming units (SFU) were distinguished to identify the responses in a first line screening process. Individual peptide-specific CD4+ T cell responses were subsequently identified using functional flow cytometric analysis to detect the production of IFNγ, IL-2, IL-21, MIP-1β and TNF. Out of the 64 peptides, 47 (73%) generated a CD4+ T cell response in at least one patient. There was a clear skewing of CD4+ T cell responses toward Gag and Nef peptides (Figure 2A). The percentage and number of individuals recognising each peptide is stated in Table S1. Each subject recognised a median of 5 peptides (IQR: 2.75–6.00) with a maximum of 12 recognised peptides, which was observed in one patient (Figure 2B). Within the cohort, almost all of the peptides derived from the Gag (100%) and Nef (93%) regions and the majority of the peptides from the Pol (66%) and Tat (75%) regions were immunogenic, while only a limited number of peptides located in the Env (33%) region generated responses (Figure 2C).

Although a majority of peptides within the different HIV protein regions were recognised, the fraction of tested peptides in each region that elicited responses differed (one-way ANOVA: P<0.001, n  = 38; Figure 2D). Gag and Nef peptides were more broadly reactive and induced responses in a significantly higher fraction of individuals than did peptides in other protein regions. Gag- and Nef-specific CD4+ T cell responses were detected at least 4.4 (P<0.001, n  = 38) and 2.5 (P<0.01, n  = 38) times more frequently than responses against the Pol, Env, and Tat peptides (Figure 2D). There were no statistically significant differences in the frequencies of the responses between peptides in the Pol, Env, and Tat regions. The distribution of the responses is illustrated in Figure S1. Interestingly, a significant inverse correlation was observed between the number of targeted Nef and Gag peptides (r  =  −0.36, P  = 0.029, exact permutation test, n  = 38), implicating that CD4+ T cell responses in the majority of the cohort were dominated by either broad Gag- or Nef-specific responses.

### HLA Restriction for Peptides Inducing CD4+ T Cell Responses using Prediction Tools

Binding to one or more of the donors HLA molecules is a prerequisite for a peptide to induce a T cell response. An HLA restriction analysis was performed for the observed CD4+ T cell responses. The analysis was limited to the subset of alleles where accurate prediction methods exist (HLA-DR covered by *NetMHCIIpan-2.0*, and 5 DP and 6 DQ alleles covered by *NetMHCII-2.2*). Using a binding threshold of 500 nM, we found that 160 of the 172 (93%) positive CD4+ T cell responses in donors with known HLA type could be explained by predicted binding to one or more of the donor HLA class II molecules. Several of the epitopes were predicted to be highly promiscuous and the median number of predicted restriction elements per peptide was 5. The highest number of restrictions (40) was found for the peptide VDRFYKTLRAEQASQ earlier reported to have a high cross-reactive binding capacity [7]. Likewise, were the HLA-DRB1*0101 and HLA-DRB1*1501 molecules found to be the most prevalent restriction elements predicted to bind 31 and 32, respectively, of the 46 experimentally validated epitopes in donor with annotated HLA types. Details on the number of distinct predicted HLA restrictions for each positive peptide is included in supplementary table S1.

### Inverse Correlation between the Breadth of Gag-specific CD4+ T Cell Responses and Viral Load

**Figure 3:**
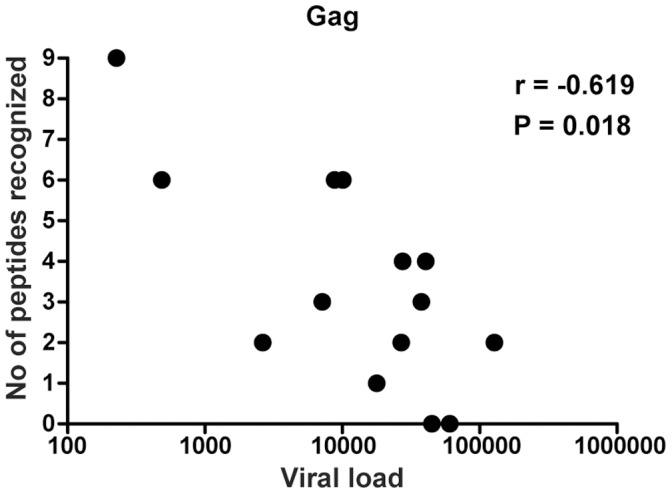
Association between the breadth of Gag-specific CD4+ T cells and HIV viremia. Correlation between CD4+ T cells targeting multiple Gag peptides and viral load in viremic subjects using the Spearman non-parametric test. Similar correlation and significance was obtained excluding subject THS-15, who had low-grade viremia and documented poor treatment adherence.

Next, clinical parameters were associated with the breadth of peptide-specific CD4+ T cell responses. Figure 3 gives the result of such an analysis and demonstrates that broad Gag targeting is significantly and inversely correlated with viral load in viremic individuals (r  =  −0.619, P  = 0.018, n  = 14). The correlation was not maintained when looking at recognised peptides from all of the HIV protein regions (data not shown). No correlations between broad peptide targeting and CD4+ T cell counts were identified.

### Functional Discrepancies between CD4+ T Cells Targeting Different HIV Proteins

**Figure 4:**
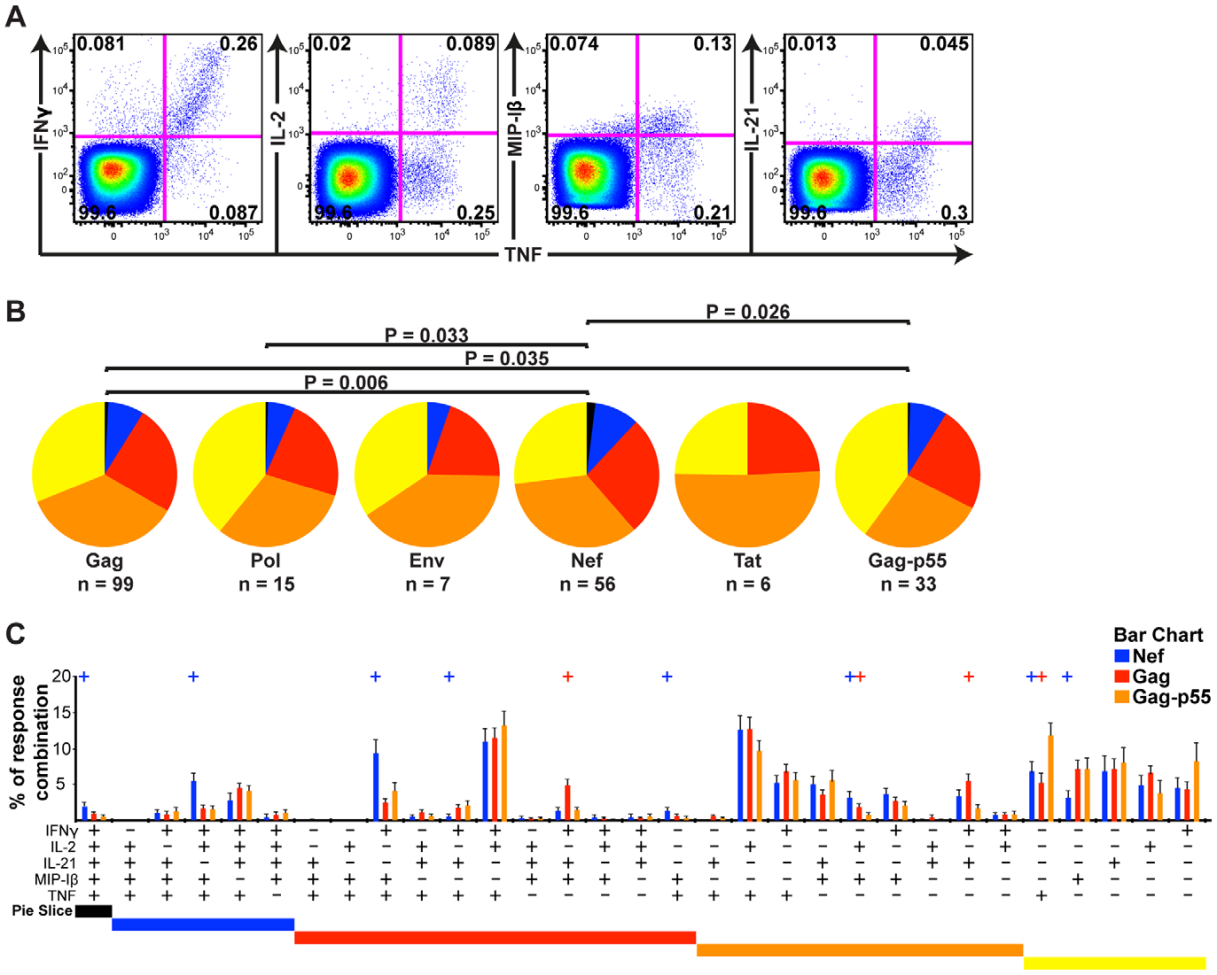
Functional discrepancies of CD4+ T cells targeting predicted peptides of different HIV protein regions. (A) Representative flow cytometric plots showing the pattern of CD4+ T cell TNF secretion along with IFNγ, IL-2, MIP-1β and IL-21 upon Gag-p55 stimulation. (B) Pie charts representing the fraction of functions upregulated by the various HIV protein-specific CD4+ T cells; from one (yellow) up to five functions (black) are illustrated by the colour-scale. Permutation tests were performed to compare the differences between the pie charts. (C) Frequency comparison of the functions of Nef-specific (blue), Gag-specific (red) and Gag-p55-specific (orange) CD4+ T cells for each of the 31 functional combinations. Bars represent the means of a functional combination, and upper whiskers show SEM. Significant differences between the bars for Gag-p55 and Gag or Nef are represented by +, which indicates P<0.05 using Student’s t-test.

While Gag and Nef peptides induced CD4+ T cell responses more frequently than the Pol, Env and Tat peptides, the magnitudes of the detected responses were not significantly different (one-way ANOVA: P>0.05, n  = 38; data not shown). The median magnitude (% of tot CD4+ T cells) and IQRs of the responses against each peptide is depicted in Table S1. The similar magnitudes of the responses of HIV-specific CD4+ T cells targeting the predicted peptides from the diverse protein regions allowed us to study functional discrepancies between the different HIV proteins. A boolean gating principle was applied to create combinational events of IFNγ, IL-2, IL-21, MIP-1β and TNF in order to investigate functional diversities (Figure 4A). By measuring the diversity of polyfunctionality, we found that immunogenic Nef peptides (n  = 56) led to the production of more functional combinations of these cytokines than did the immunogenic Gag (P  = 0.006, n  = 99), and Pol (P  = 0.033, n  = 15) peptides (Figure 4B). In addition, the immunogenic Gag peptides induced a more functionally diverse response compared to the set of overlapping pool of Gag-p55 peptides (P  = 0.035) that was used as one of the positive controls (Figure 4B). More precisely, the CD4+ T cells that targeted the predicted Nef and Gag epitopes expressed three functional combinations more frequently than cells recognising the overlapping Gag-p55 peptide pool. The Gag-p55 peptide pool-specific CD4+ T cells frequently exhibited monofunctional responses, particularly TNF and, more modestly, MIP-1β (Figure 4C). There were no significant functional differences observed for the positive Env and Tat peptides when compared to the other protein regions, probably due to the lower number of responses. Taken together, these data suggests that the HLA class II restricted peptides identified in this study generate an improved polyfunctionality compared to overlapping peptides.

## Discussion

The tremendous variability of the human immune system and the HIV genome represents a major challenge in identifying broadly reactive, peptide-specific CD4+ T cell responses in diverse populations. The utilisation of bioinformatics to identify T cell responses to HIV infections has increased in the last few years [14], [19], [20]. For peptide prediction, the *in silico* prediction methods *NetMHCIIpan* and *NetMHCII* developed to predict HLA class II restricted peptide binding have proven to be among the best methods available [21]. Here, we integrated these prediction methods with a novel algorithm, *PopCover*, to select HLA-DR- and –DQ-restricted HIV peptides with broad HLA allelic and HIV genomic coverage. *PopCover* integrates HLA binding predictions with information about HLA allelic prevalence in a given population and selects pools of peptides that, in concert, will provide broad coverage of both the pathogen genome (*i.e.* HIV subtypes and strains) and HLA diversity. Using this approach, 64 top-scoring peptides were selected to identify broadly reactive HIV-specific CD4+ T cell responses. A majority of the predicted peptides were found to be immunogenic. The Gag- and Nef-specific CD4+ T cell responses dominated. Targeting of Gag peptides was found to be associated with a lower viral load in viremic individuals. Importantly, a vast majority of the peptides were predicted to be restricted to one or more of the donor’s HLA class II alleles.

At least one of the predicted HLA class II binding peptides generated a response in 37 out of 38 individuals. Freshly isolated PBMCs were used to assay the peptide immunogenicity as it has been demonstrated to the increase the detection sensitivity of HIV-specific CD4+ T cell responses [22]. In total 47 (73%) of the predicted peptides generated a CD4+ T cell response. Although a majority of the peptides were immunogenic, CD4+ T cell responses against Gag and Nef occurred more frequently, which recapitulates other comprehensive studies [7], [8]. Previously, through a comprehensive analysis that used 410 overlapping peptides spanning the entire HIV subtype B genome, Kaufmann *et al.* [7] detected eight peptides (1.8%) that were recognised in more than 25% of the study cohort (n  = 36). Using only 64 peptides, we identified eight immunodominant peptides (12.5%) that generated CD4+ T cell responses in at least 20% of our subjects. Similarly, when the frequencies of peptide-specific responses per patient are compared between the studies, our approach was 4.4-times more likely to identify peptide-specific responses. Kaufmann *et al.* detected responses against 1.7% of the tested epitopes (range 0–8.3%), while our approach detected 7.5% (range 0–17%).

Most of the peptides described as immunodominant in the present study have been described previously. For example, the most immunogenic of our predicted peptides (VDRFYKTLRAEQASQ – Gag-VQ15) is located within a conserved part of the Gag-p24 region important for capsid oligomerization [23]. Gag-VQ15 has been identified as the most frequently targeted peptide by CD4+ T cells in subtype B infected subjects [7] potentially due to its restriction to multiple HLA-DR alleles (http://www.hiv.lanl.gov/). The second most immunogenic peptide, EVLMWKFDSRLAFHH – Nef-EH15, overlapped with another peptide sequence (10 amino acids), previously shown to bind to a diverse set of HLA-DRB1 and HLA-DRB5 alleles (http://www.hiv.lanl.gov/). However, other immunodominant peptides that generated responses in >20% of our study subjects, (*e.g.* Nef-FR15 and Gag-GL15) have not been described previously as broadly immunogenic peptides. Out of 47 peptides generating CD4+ T cell responses, only 75% (35) shared a 9****mer core (9 consecutive identical amino acids) with one or more known HLA class II-restricted epitopes from the Los Alamos HIV database. According to this criteria, 25% of the peptides generating responses are thus of novel character.

Despite the fact that HIV preferentially infects HIV-specific CD4+ T cells [24], early preservation of these cells most probably represent a crucial mechanism for viral control [25], [26]. Specific targeting of the Gag peptides by CD4+ T cells has been associated with lower viremia in both adults [27] and children [28], [29]. A recent study has demonstrated an inverse correlation between viral load and the number of Gag peptides targeted by CD4+ T cells [9]. Our data in viremic subjects confirm this finding, suggesting that broadly reactive Gag-specific CD4+ T cell responses could have an impact on HIV disease progression. However, whether the frequent targeting of Gag peptides is the cause or the consequence of the reduced viremia remains to be clarified.

The functional characteristics of HIV-specific T cells have been proposed to correlate to the viremic state of the individual [30]–[33]. In our cohort, we found differences in the polyfunctionality of the CD4+ T cells targeting the different HIV protein regions. Of note, it was demonstrated that the CD4+ T cell responses induced by the overlapping Gag-p55 peptide pool were skewed toward a more limited functional profile compared to the predicted, broadly reactive Gag and Nef peptides. These variations should be considered when interpreting HIV-specific CD4+ T cell responses. Additionally, the Nef-specific CD4+ T cell responses showed the highest degree of polyfunctionality. However, this might have been linked to the tendency of increased Nef targeting within the treated individuals (unpublished observations).

In summary, we believe to have demonstrated that computational predictions in general provide a powerful tool for studying peptide-targeting in natural infection and vaccine trials. Particularly, we have shown that the *PopCover* method is a simple yet highly effective method for selection of peptide-pools covering both pathogen and host immune system diversity. In the present study, it was shown that 64 predicted HLA class II-binding peptides could be used to reveal a pattern of CD4+ T cell immunodominance similar to that yielded by comprehensive analysis spanning the entire HIV genome. In addition, both the functional properties and breadth of specific CD4+ T cell responses in correlation with viremia revealed patterns similar to previous comprehensive studies. In conclusion, the utilization of peptide selection strategies, like *PopCover,* might be fruitful in the future design and evaluation of HIV vaccines.

## Materials and Methods

### Ethics Statement

This study was approved by the Regional Ethical Council (Stockholm, Sweden 2009/1592-32) and all study participants provided informed consent. Oral and written information was provided. Oral informed consent was documented in the patients’ journal. Written informed consent was not provided as HIV infected individuals, especially from non-literate and developing societies, may view the use of written consent documents as a binding document, not protecting the study participant. This perception can undermine trust and may risk coercing people to sign such documents.

### Subjects

A total of 38 HIV infected individuals were recruited from the Karolinska University Hospital and Stockholm South General Hospital, Stockholm, Sweden (Table 1). Patient inclusion was based on the single criteria of a CD4+ T cell count >300 cells/mm^3^ to ensure detection of HIV-specific CD4+ T cell responses. Of the included patients, 25 were treated (viral load <50 copies/mL, except for individual THS-15) and 13 untreated (viral load >50 copies/mL).

### Prediction and Selection of Peptides

The peptide selection was made from a dataset containing between 395 and 424 (depending on the given HIV protein) full-length HIV genomic sequences covering 50 subtype A, 104 subtype B, 156 subtype C, 40 subtype D and 46 CRF_01AE strains. For each HIV amino acid sequence the HLA peptide binding prediction methods *NetMHCII* [16] and *NetMHCIIpan* [17], [18] were applied to predict 15****mer peptide-binders to a set of HLA class II alleles. Four prevalent HLA-DQ alleles (DQA1*0101-DQB1*0501, DQA1*0301-DQB1*0302, DQA1*0401-DQB1*0402, and DQA1*0501-DQB1*0301), 38 HLA-DRB1 alleles and HLA-DRB3*01∶01, HLA-DRB4*01∶01 and HLA-DRB5*01∶01 were included in the analysis. A predicted IC50 value of 500 nM was applied to define peptide-binders. Next, peptides were selected using a novel algorithm, *PopCover*, to maximize broad allelic and pathogen coverage. The scoring function used to select peptides was defined as
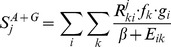
where *R^j^_ki_* is 1 if peptide *j* is present in genome *i* and is presented by allele *k*, *E_ik_* is the number of times allele k has been a target by peptide in genome *i* by the already selected set of epitopes, *g_i_* is the genomes frequency which is normally set to being uniform, *f_k_* is the allelic frequency of allele *k*, and β is a tuneable constant set to penalize un-targeted pathogen-HLA pairs. The scoring function is iteratively applied to all predicted peptide binders from the pathogen proteomes/protein to the alleles included in the study, and the highest scoring peptide is selected in each selection round.

In total, the number of unique predicted 15****mer HLA class II binders for each protein set was 125,926 (Env), 31,848 (Gag), 20,962 (Nef), 42,749 (Pol), and 5,608 (Tat). From this set of predicted peptides, the *PopCover* algorithm, using a value of β  = 0.05, was applied to select the final set of 64 (15 Gag, 15 Pol, 15 Env, 15 Nef, and 4 Tat) top-scoring peptides. When the *PopCover* algorithm is initialized, the variable *E_ik_* in the *PopCover* scoring function is zero for all alleles and genomes, and the first peptide selected is the peptide that is present in most HIV genomes and binds to the set of HLA alleles with the highest allelic frequency sum. When the next peptide is selected, the score for targeting an allele and genome already covered by the first peptide is lowered by a factor (1+ β)/β  = 21 (ignoring differences in allelic frequency and assuming equal genomic coverage) and the selection will hence focus on identifying peptides that target previously un-targeted HLA alleles and genomes.

### HIV Extraction, Sequencing and Subtyping

RNA or DNA was extracted from plasma or PBMCs using the RNeasy Lipid Tissue Mini Kit or DNA kit (Qiagen). The RNA was used to generate cDNA, whereas the DNA was directly used for PCR. A nested PCR for the HIV *gag* gene (p17 and p24) was performed as previously described [14], [34] with some primer modification.

Sequencing was performed using the BigDye Terminator Version 3.1 Cycle Sequencing Kit (Applied Biosystems) and detected in the ABI PRISM 3130*xl* Genetic Analyzer (Applied Biosystems). Sequences were imported and manually edited using Sequencher software (Gene Codes). The *gag* gene sequences and recommended subtype reference sequences (www.hiv.lanl.gov) were used to construct neighbour-joining phylogenetic trees using the MEGA 4 software [35]. Phylogenetic tree analysis was used to determine the subtype of the *gag* gene sequence and to facilitate detection of possible PCR contamination and sample mix-up. Subtyping results were also compared with online subtyping by Standford and REGA tools. The HIV *gag* sequences have been submitted to GenBank and given the accession numbers JQ229725 - JQ229761.

### HLA Typing

HLA-A, -B, -C, -DPA1, -DPB1, -DQA1, -DQB1, and -DRB genes were amplified by PCR from patient DNA samples, using gene specific primers for each HLA gene. PCR amplification was performed according to Luo *et al.* [36], using Taq DNA polymerase (Invitrogen). PCR products were examined by agarose gel electrophoresis and purified using the Agencourt AMPure PCR magnetic bead purification system (Beckman Coulter). The purified PCR products were sequenced using an ABI PRISM BigDye Terminator Cycle sequencing system (Applied Biosystems) and analyzed with an ABI Prism 3130 or 3700 Genetic Analyzer (Applied Biosystems). HLA genotyping was performed using computer software based on a Taxonomy-Based Sequence Analysis method [36], [37].

### Peptides

The synthesized peptides (n  = 64) were all 15-mers with purity greater than 70% (Genscript). All peptides were used at a final concentration of 2 µg/mL. As positive controls, peptide pools consisting of HCMV pp65 and HIV Gag-p55 (15-mers overlapping by 11 amino acids) (JPT technologies) and Staphylococcal enterotoxin B (SEB) (Sigma Aldrich) were obtained with >70% purity. Overlapping peptides were added to a final concentration of 1 µg/mL whereas SEB was used at a final concentration of 2.5 µg/mL.

### ELISPOT

Fresh PBMCs were isolated from whole blood collected in heparin tubes by Hypaque-Ficoll (GE Healthcare) gradient centrifugation and washed in RPMI 1640 Glutamax (Invitrogen), supplemented with 10% FCS (Invitrogen), 50 IU/mL penicillin and 50 µg/mL streptomycin, and 10 mM HEPES (R10). The IFNγ ELISPOT has been described elsewhere [14].

### Conjugated Antibodies

The following directly conjugated antibodies were used to detect antigen-specific T cell responses: CD3–APC-H7 (SK7), CD4–V450 (RPA-T4), CD8–Amcyan (SK1), IFNγ–FITC (B27), TNF–PE-Cy7 (MAb11), IL-2–APC (MQ1-17H12), MIP-Iβ–PerCP-Cy5.5 (D21-1351) and IL-21–PE (3A3-N2.1) (BD Biosciences).

### Intracellular Cytokine Staining (ICS)

Directly following the ELISPOT analysis each single predicted peptide, from the peptide pools generating responses in the ELISPOT experiments, were tested individually using ICS [38]. Fresh PBMC counts and viability were assessed using a Nucleocounter (ChemoMetec A/S) and resuspended in R10 to a concentration of 5×10^6^ cells/mL. U-bottom plates were plated with predicted peptides, positive controls (p55, CMV and SEB) or medium alone (negative controls) together with 5×10^5^ PBMCs/well. Brefeldin A (Sigma Aldrich) was supplemented (5 µg/mL) after 1 hr to each well and further incubated at 37°C for 11–13 hrs.

The following day, cells were transferred to V-bottom plates, washed and stained with CD8 for 20 min at 4°C. Cytofix and Perm2 (BD Biosciences) were added to fix and permeabilize the cells. After further washing, intracellular staining with CD3, CD4, IFNγ, TNF, IL-2, MIP-Iβ and IL-21 was performed for 20 min at −4°C. Cells were washed and resuspended in PBS containing 1% paraformaldehyde PFA. All flow cytometric analyses were conducted within 5 hrs.

### Flow Cytometric Analyses

PBMCs were analysed on a standardized 8 colour CantoII (BD Biosciences) where minimally 150.000 total events were collected (typically around 250.000 total events) per run. Data compensation was performed using antibody capture beads (BD Biosciences) separately stained with each single antibody used in the panel. Flow cytometric gating analyses was performed using FlowJo 8.6.6 (Treestar). Representative flow cytometric plots demonstrate the gating procedure in Figure S2A and the subsequent CD4+ T cell gating for detection of two different peptide-specific responses (Figure S2B). For all patients, two negative controls were used.

Based on two negative controls, we used two criterions to define a positive response, where either needed to be fulfilled, First, the peptide-specific CD4+ T cell responses were considered positive if the frequency of responding cells was twice the average negative background and over 0.02% of all CD4+ T cells for a single cytokine. Secondly, to increase detection sensitivity in borderline cases or when background was high, we used an additional criterion to distinguish positive responses by plotting different cytokines on the *x*- and *y*-axis [39]. If the frequency of double positive cells for any cytokine combination was four times higher than the average negative background, a response was considered positive.

### Statistics

Experimental variables between two groups of individuals were analyzed using Mann-Whitney U test and Wilcoxon matched-pairs rank test. One-Way ANOVAs followed by Kruskal-Wallis non-parametric Dunn’s multiple comparison tests were used to analyze three groups or more. Correlations were assessed using non-parametric Spearman rank tests. Statistical analyses were performed using GraphPad Prism 5.0 (GraphPad Software). All pie charts were analyzed by permutation tests using the data analysis program SPICE version 5.2009, provided by Mario Roederer, VRC, NIAID, NIH [40].

## Supporting Information

Figure S1 **Distribution of peptide targeting within different HIV protein regions.** Scatter plots showing the fraction of tested peptides within different HIV protein regions that generated CD4+ T cell responses per individual (median and IQR).
(TIF)

Figure S2 **Gating principle to distinguish HIV-specific T cell responses.** (A) Flow cytometric plots illustrating the gating strategy used to distinguish a pure CD4+ and CD8+ T cell population. The plots are for patient THS-31 (B) the negative control (left plots) and two Gag-specific CD4+ T cell responses against peptides FSPEVIPMFSALSEG – Gag-FG15 (middle plots) and RWIILGLNKIVRMYS – Gag-RS15 (right plots). The threshold for a CD4+ T cell responses was set to be twice the negative background for any cytokine and >0.02% of the total CD4+ T cell frequency. As illustrated in the top right plot, Gag-RS15 generated a positive CD4+ T cell response well over the threshold while Gag-FG15 induced a response just below borderline for TNF. To distinguish whether there was an actual response against Gag-FG15, TNF versus IL-2 plots were constructed (bottom rows). The quadrant gating shows that TNF+IL-2+ cells were over nine times the frequency of the negative control. This was more than four times the cut off (second criteria) and was thus interpreted as a positive response.
(TIFF)

Table S1 **Peptide sequences and percentages of responses.** Detailed information on the peptides sequences, pool configuration, and measured T cell response.
(PDF)
