# Supplementary figures and images for: Characterization of HIV-Specific CD4+ T Cell Responses against Peptides Selected with Broad Population and Pathogen Coverage
Source: PLoS One. 2012 Jul 5;7(7):e39874. doi: 10.1371/journal.pone.0039874 (PMC3390319; doi:10.1371/journal.pone.0039874)

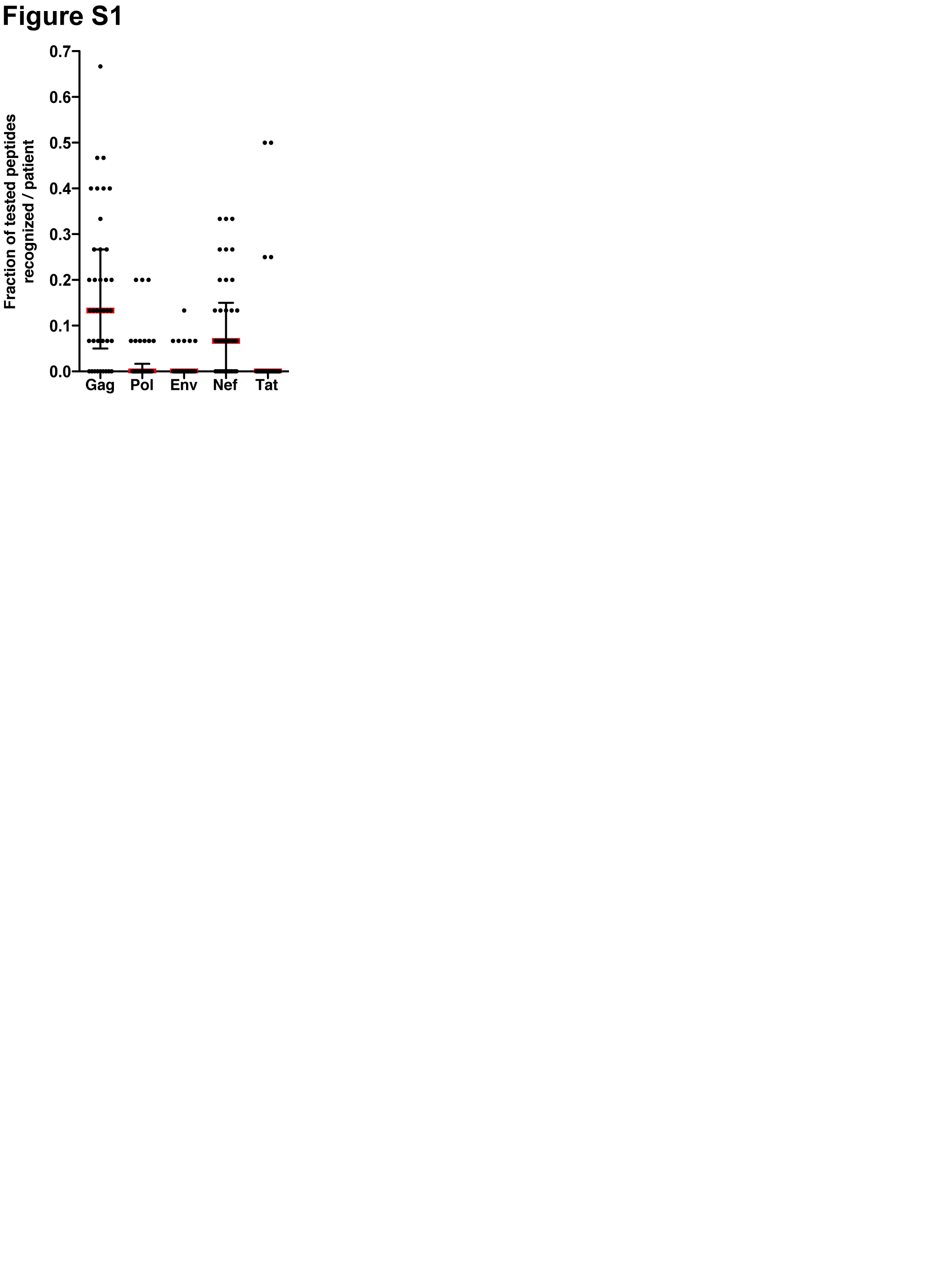

Supplement: Figure S1 — Distribution of peptide targeting within different HIV protein regions. Scatter plots showing the fraction of tested peptides within different HIV protein regions that generated CD4+ T cell responses per individual (median and IQR). (TIF) [file pone.0039874.s001.tif]

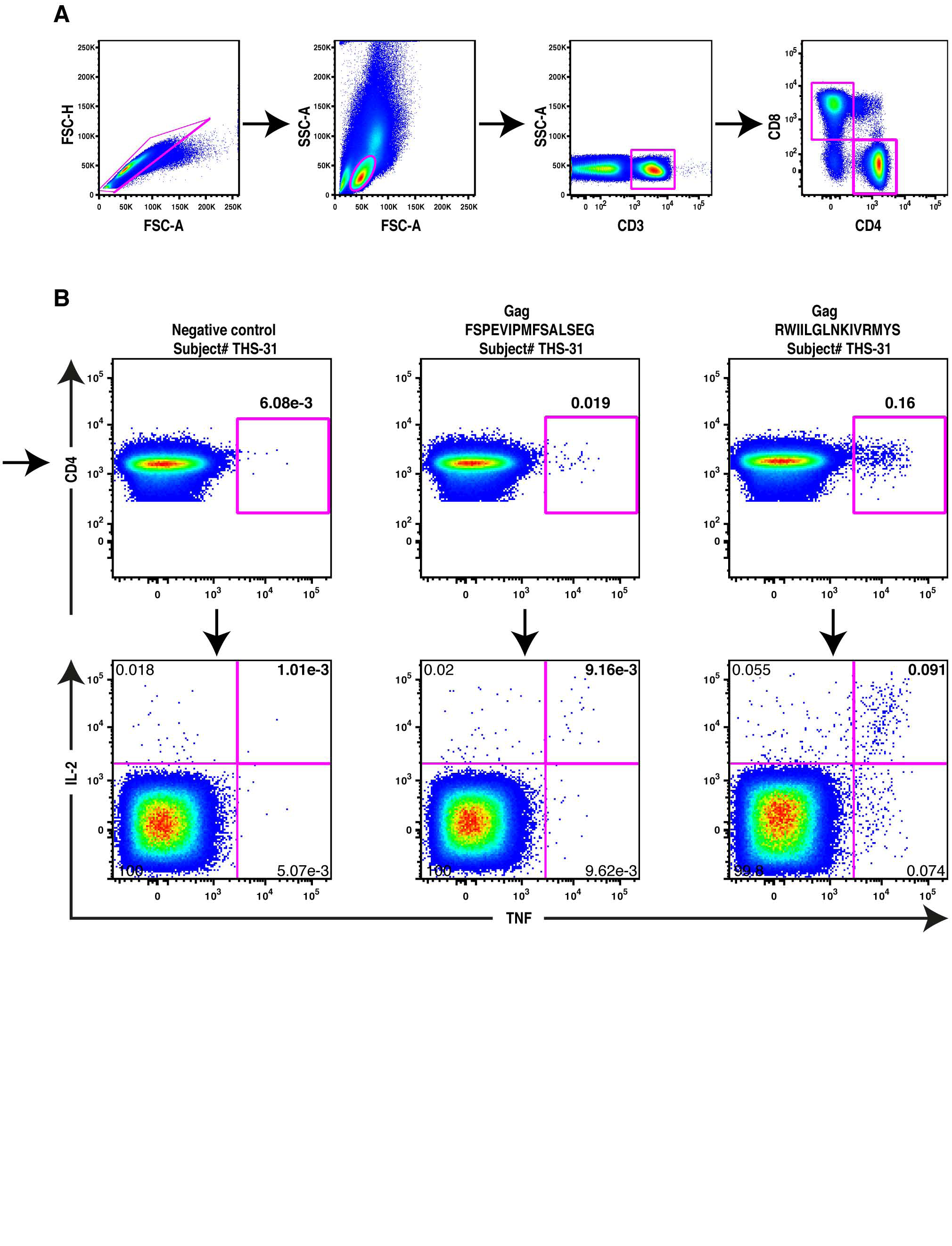

Supplement: Figure S2 — Gating principle to distinguish HIV-specific T cell responses. (A) Flow cytometric plots illustrating the gating strategy used to distinguish a pure CD4+ and CD8+ T cell population. The plots are for patient THS-31 (B) the negative control (left plots) and two Gag-specific CD4+ T cell responses against peptides FSPEVIPMFSALSEG – Gag-FG15 (middle plots) and RWIILGLNKIVRMYS – Gag-RS15 (right plots). The threshold for a CD4+ T cell responses was set to be twice the negative background for any cytokine and >0.02% of the total CD4+ T cell frequency. As illustrated in the top right plot, Gag-RS15 generated a positive CD4+ T cell response well over the threshold while Gag-FG15 induced a response just below borderline for TNF. To distinguish whether there was an actual response against Gag-FG15, TNF versus IL-2 plots were constructed (bottom rows). The quadrant gating shows that TNF+IL-2+ cells were over nine times the frequency of the negative control. This was more than four times the cut off (second criteria) and was thus interpreted as a positive response. (TIFF) [file pone.0039874.s002.tif]
