# Supplementary material for: Characterization of HIV-Specific CD4+ T Cell Responses against Peptides Selected with Broad Population and Pathogen Coverage
Source: PLoS One. 2012 Jul 5;7(7):e39874. doi: 10.1371/journal.pone.0039874 (PMC3390319; doi:10.1371/journal.pone.0039874)
Supplement: Table S1 — Peptide sequences and percentages of responses. Detailed information on the peptides sequences, pool configuration, and measured T cell response. (PDF) [file pone.0039874.s003.pdf]

Table S1: Peptide sequences and percentages of responses

Peptide No	Peptide sequence	Peptide abbrevation	ELISPOT Pool	% of individuals with CD4+ response	Number of individuals with CD4+ responses	Median frequency§ & IQR of CD4+ responses	Number of predicted HLA restrictions
Gag							
1	RWIILGLNKIVRMYS	Gag-RS15	E	13.2	5	0.034 (0.026-0.11)	11
2	GLNKIVRMYSPVSIL	Gag-GL15	F	15.8	6	0.067 (0.044-0.20)	15
3	GLNKIVRMYSPTSIL	Gag-GL15_T12	F	26.3	10	0.048 (0.034-0.11)	22
4	ILGLNKIVRMYSPVS	Gag-IS15	F	21.1	8	0.027 (0.024-0.084)	16
5	PSHKARVLAEAMSQA	Gag-PA15	F	15.8	6	0.048 (0.029-0.094)	8
6	VDRFYKTLRAEQASQ	Gag-VQ15	G	50	19	0.032 (0.020-0.043)	40
7	IVRMYSPVSILDIKQ	Gag-IQ15	G	21.1	8	0.035 (0.028-0.040)	26
8	NWMTDILLVQNANPD	Gag-ND15	G	13.2	5	0.034 (0.019-0.043)	6
9	FSPEVIPMFSALSEG	Gag-FG15	G	23.7	9	0.042 (0.030-0.049)	13
10	WIILGLNKIVRMYSP	Gag-WP15	H	10.5	4	0.056 (0.037-0.09)	8
11	DVYKRWIILGLNKIV	Gag-DV15	H	15.8	6	0.039 (0.019-0.13)	11
12	EVKNWMTDTLLVQNA	Gag-EA15	H	5.3	2	0.065 (0.047-0.083)	2
13	YMIKHIVWASRELER	Gag-YA15	H	10.5	4	0.033 (0.014-0.057)	9
14	IYKRWIILGLNKIVR	Gag-IR15	I	13.2	5	0.029 (0.017-0.032)	10
15	ESFRDYVDRFYKTLR	Gag-ER15	I	7.9	3	0.040 (0.011-0.058)	4
Pol							
16	IPEWEFVNTPPLVKL	Pol-IL15	I	7.9	3	0.038 (0.020-0.047)	8
17	SWTVNDIQKLVGKLV	Pol-SV15	I	2.6	1	0.091 (0.091-0.091)	1
18	HLKTAVQMAVYIHNF	Pol-HF15	J	7.9	3	0.050 (0.030-0.065)	5
19	KWEFVNTPPLVKLWY	Pol-KY15	J	2.6	1	0.034 (0.034-0.034)	2
20	QYALGIIQAQPDRSE	Pol-QE15	J	0	0	0 (0-0)	NA
21	ISKFRVYYRDSRDPI	Pol-II15	J	2.6	1	0.10 (0.10-0.10)	2
22	NFPISPIETVPVKLR	Pol-NR15	K	2.6	1	0.089 (0.089-0.089)	1
23	PSGLKKKKSVTVLDM	Pol-PM15	K	2.6	1	0.032 (0.032-0.032)	1
24	VTSGYIEAEVIPAET	Pol-VT15	K	2.6	1	0.11 (0.11-0.11)	2
25	IWQLDCTHLEGKVIL	Pol-IL15(2)	K	0	0	0 (0-0)	NA
26	QGWKGSPAIFQSSMT	Pol-QT15	L	0	0	0 (0-0)	NA
27	EWEFVNTPPLVKLWY	Pol-EY15	L	5.3	2	0.11 (0.044-0.18)	6
28	VHDIQKLVGKLNWAS	Pol-VS15	L	2.6	1	0.068 (0.068-0.068)	1
29	KSVTVLDVGDAYFSV	Pol-KV15	L	0	0	0 (0-0)	NA
30	HTAGLKKKKSVTVLD	Pol-HD15	M	0	0	0 (0-0)	NA
Env							
31	SVINRVRQGYSPLSF	Env-SF15	M	0	0	0 (0-0)	NA
32	PVVSTQLLLNGSLAE	Env-PE15	M	2.6	1	0.073 (0.073-0.073)	2
33	QYNLLRAIEAQQHLL	Env-QL15	M	0	0	0 (0-0)	NA
34	FVVLSIVNRVRQGYS	Env-FS15	N	5.3	2	0.065 (0.014-0.12)	5

35	AVERYLKDQQLLGIW	Env-AW15	N	0	0	0 (0-0)	NA
36	RTFIMIVGGLIGLRI	Env-RI15	N	2.6	1	0.087 (0.087-0.087)	3
37	WVTVYYGVPVWKEAE	Env-WE15	N	0	0	0 (0-0)	NA
38	AVERYLRDQQLLGIW	Env-AW15_L7	O	0	0	0 (0-0)	NA
39	RVVEREKRAVGLLGA	Env-RA15	O	0	0	0 (0-0)	NA
40	LTEGEIIIRSENLTN	Env-LN15	O	0	0	0 (0-0)	NA
41	TVWGIKQLQARVLAV	Env-TV15	O	0	0	0 (0-0)	NA
42	WVTVYYGVPVWKDAE	Env-WE15_D13	P	2.6	1	0.020 (0.020-0.020)	2
43	FYAARKIIGDIRQAH	Env-FH15	P	0	0	0 (0-0)	NA
44	GIKQLQARVLAVERY	Env-GY15	P	5.3	2	0.022 (0.016-0.028)	5
45	IVQQQSNLLRAIKAQ	Env-IQ15	P	0	0	0 (0-0)	NA
Nef							
46	FPVRPQVPLRPMTYR	Nef-FR15	B	21.1	8	0.031 (0.024-0.061)	5
47	AVDLSHFLKEKGGLM	Nef-AM15	B	13.2	5	0.067 (0.043-0.090)	6
48	GWPFKLVPVDPREVQ	Nef-GQ15	B	5.3	2	0.022 (0.013-0.031)	3
49	GWFLKEKGGLDGLIY	Nef-GY15	B	13.2	5	0.017 (0.011-0.027)	4
50	EVLMWKFDSRLAFHH	Nef-EH15	C	28.9	11	0.038 (0.029-0.053)	22
51	SWFLKEKGGLEGLIY	Nef-SY15	C	10.5	4	0.060 (0.049-0.068)	1
52	EVLMWKFDSRLALTH	Nef-EH15_L12_T13	C	23.7	9	0.039 (0.033-0.050)	22
53	KWSKNRIVGWPAVRE	Nef-KE15	C	2.6	1	0.025 (0.025-0.025)	1
54	QVPLRPMTYKGALDL	Nef-QL15	D	5.3	2	0.049 (0.020-0.079)	0
55	GKWSKSSIVGWSAVR	Nef-GR15	D	5.3	2	0.079 (0.043-0.12)	4
56	RWEFDSSLARRHLAR	Nef-RR15	D	2.6	1	0.067 (0.067-0.067)	2
57	EVLVWRFDSRLAFHH	Nef-EH15_V4_R6	D	7.9	3	0.026 (0.024-0.056)	4
58	GWCFKLVPVEPEEVE	Nef-GE15	E	2.6	1	0.028 (0.028-0.028)	NA (donor not typed)
59	GWCFKLVPVDPDEVE	Nef-GE15_D10_D12	E	7.9	3	0.060 (0.036-0.11)	5
60	GLDGLIYSKKRQEIL	Nef-GL15	E	0	0	0 (0-0)	NA
Tat							
61	ITKALGISYGRKKRR	Tat-IR15	A	2.6	1	0.086 (0.086-0.086)	3
62	CFLKKGLGISYGRKK	Tat-CK15	A	0	0	0 (0-0)	
63	SYHCLVCFQTKGLGI	Tat-SI15	A	7.9	3	0.038 (0.016-0.067)	4
64	DHQNLISKQPLPRTQ	Tat-DQ15	A	5.3	2	0.035 (0.016-0.053)	3

§ % of total CD4+ T cells
